# Athletes beyond sex categories: gender inclusivity, law and sport in India

**DOI:** 10.3389/fspor.2026.1767095

**Published:** 2026-04-17

**Authors:** Tarun Tarun, Anamika Shukla, Niyati Pandey

**Affiliations:** Gujarat National Law University, Gandhinagar, India

**Keywords:** constitutional rights, governance, inclusion, Indian sports, safe sports, trans athletes

## Abstract

India’s sporting ecosystem remains deeply entrenched in binary classifications of sex, despite the country’s long-standing cultural recognition of gender diversity and its progressive constitutional jurisprudence on gender identity. This paper examines India’s engagement with transgender, non-binary, and sex-variant athletes, highlighting the disconnect between legal recognition and sporting governance. While landmark judicial decisions such as NALSA vs. Union of India (2014) have affirmed the right to self-determined gender identity and constitutional guarantees of dignity, equality and non-discrimination, these principles have yet to be meaningfully operationalised within Indian sport. Through a socio-legal analysis of prominent Indian case studies of Anaya Banger, Aryan Pasha, Dutee Chand, Shanthi Soundararajan, and Nandini Agasara, the paper illustrates how athletes who fall outside normative sex-based classifications face invasive scrutiny, regulatory exclusion, privacy violations, and social stigma. These cases demonstrate how sports authorities frequently conflate transgender identity with intersex variations and natural biological diversity, resulting in inappropriate eligibility standards and coercive medical practices. The paper situates these experiences within broader international developments, including fragmented global sporting regulations. The analysis further explores India's constitutional framework, statutory protections under the Transgender Persons (Protection of Rights) Act, and relevant international human rights obligations, arguing that the principal barrier to inclusion is not the absence of rights but the failure to implement them within sport-specific governance. The paper concludes by proposing pathways for reform, including the adoption of uniform national guidelines, affirmative support measures, institutional sensitisation, and evidence-based policy design. It argues that inclusive sport is not merely a matter of competitive fairness, but a measure of democratic belonging, dignity, and substantive equality in practice.

## 1 Introduction

The debate surrounding sex and gender has arguably reached conceptual clarity in non-sporting domains. Sex is generally understood to refer to biological and physical attributes traditionally classified as male or female, whereas gender encompasses social, cultural, psychological, and identity-based dimensions (1). In most areas of public life like education, employment, healthcare, and legal recognition, gender inclusivity has increasingly been foregrounded. Sport, however, remains a notable exception. Competitive sport continues to be governed predominantly by sex-based classifications, maintaining rigid male and female categories justified on grounds of fairness safety and physical difference.

This tension has been partially acknowledged at the international level. The International Olympic Committee's (2) Framework on Fairness, Inclusion and Non-Discrimination on the Basis of Gender Identity and Sex Variations (2) sought to recalibrate the balance between safety, fairness, and inclusivity. However, the framework refrained from prescribing uniform eligibility rules and instead delegated decision-making authority to individual international sports federations. This decentralised approach has resulted in fragmented, inconsistent, and often opaque policies governing transgender and non-binary athletes across different sports.

A critical question that emerges, and one that this paper seeks to foreground, is whether all athletes who do not fall into the normative sports categories of male and female are necessarily transgender athletes, and if not, why they are frequently treated as such within sports governance. Athletes with intersex variations, naturally occurring hormonal differences, or non-conforming sex characteristics are often subsumed within regulatory regimes designed for transgender participation, despite having distinct biological, legal, and ethical considerations. This conflation has significant consequences, ranging from inappropriate eligibility standards to coercive medical scrutiny and exclusion from competition.

India provides a particularly compelling context for this inquiry. The subcontinent has a long history of recognising gender diversity, with references to third-gender identities found in ancient Hindu texts and long-standing social and religious practices (3, 4). Although British colonial legal regimes marginalised and criminalised these identities, post-independence India has taken meaningful steps toward legal recognition. The Supreme Court's landmark decision in (5) (hereinafter referred to as the “NALSA” judgement) affirmed the constitutional rights of transgender persons and formally recognised the “third gender” under Article 21 of the Constitution.

Despite these advances in constitutional and social recognition, Indian sport has largely remained entrenched in a binary framework. In practice, athletes who do not conform to conventional male or female classifications have been subjected to intense scrutiny, invasive and coercive gender verification practices, public stigma, and career-altering exclusions (6–9). These experiences underscore a sharp disjunction between India's evolving constitutional values and its sporting governance structures.

Against this backdrop, this paper undertakes a socio-legal analysis of key Indian case studies involving transgender and athletes not falling into normative sports categories. It examines existing legal frameworks, institutional practices, and policy gaps, and evaluates how current approaches fail to adequately address the realities of gender-diverse athletes and the steps taken towards their inclusion. Finally, the paper proposes pathways for developing inclusive, scientifically grounded, and rights-based sporting policies in India, ones that move beyond *ad hoc* decision-making and recognise the plurality of bodies and identities in sport. This paper examines India's engagement with transgender and athletes not falling into normative sports categories, with a primary focus on transgender athletes and their inclusion within the existing sports framework. Particular attention is devoted to the experiences of Aryan Pasha and Anaya Banger, whose sporting journeys illuminate contemporary challenges of participation, eligibility, and recognition faced by transgender athletes in India. At the same time, the paper also analyses the cases of Dutee Chand, Shanthi Soundararajan, and Nandini Agasara to highlight the distinct but related challenges encountered by athletes who do not fit neatly within rigid sex-based sporting classifications. Together, these cases reveal how sports governance struggles to accommodate bodies, identities, and physiologies that fall outside conventional binaries.

## 2 Assessment of India’s policy framework

### 2.1 Constitutional safeguards & international obligations

#### 2.1.1 Article 14

Indian constitution guarantees inclusion of transgender persons in society which extends to sporting ecosystem. Article 14 of the Constitution of India, ensures equality before the law. Article 14 (71) has been read expansively by the Supreme Court to prohibit both arbitrary state action and discrimination. The provision yields two complementary guarantees: (a) formal equality (equality before the law) which prevents arbitrary state action; and (b) substantive equality (equal protection of the laws) which requires reasonable classification and non-discriminatory treatment. The Court has repeatedly held that classifications must (i) be based on intelligible differentia and (ii) have a rational nexus to the object the classification seeks to achieve.^1^

The power of Article 14 ensures the prohibition of arbitrary exclusion of individuals from sport. When a state agency or a public sports authority bars an athlete from participation on the basis of gender identity without a rational, evidence-based justification, that exclusion challenges the very spirit of Article 14. The athlete may challenge such exclusion as arbitrary or unreasonable classification. For example, a rule that automatically bars all trans women from women's events, without sport-specific scientific evidence that such a ban is necessary and proportionate, risks running afoul of Article 14 requirement of a rational nexus.

Article 14 requires any eligibility rule that classifies athletes by sex/gender to be based on intelligible differentia and proportionality. A federation rule that relies on an arbitrary hormonal threshold, sans high-quality, sport-specific evidence, could be in violation of Article 14 safeguards. The CAS interim ruling in Dutee Chand emphasised the need for robust scientific proof before a regulatory classification excluding athletes is imposed which is an approach consonant with Article 14's rationality mandate.

#### 2.1.2 Article 15

On equal footing are the protections under Article 15 (72) which prohibit discrimination “on grounds of religion, race, caste, sex or place of birth” and explicitly empowers the State to make special provisions for backward or disadvantaged classes. Article 15 (4) states that “*Nothing in this article or in clause (2) of article 29 shall prevent the State from making any special provision for the advancement of any socially and educationally backward classes of citizens or for the Scheduled Castes and the Scheduled Tribes.*” On similar lines, Article 15(5) states that “*Nothing in this article or in sub-clause (g) of clause (1) of article 19 shall prevent the State from making any special provision, by law, for the advancement of any socially and educationally backward classes of citizens or for the Scheduled Castes or the Scheduled Tribes in so far as such special provisions relate to their admission to educational institutions including private educational institutions, whether aided or unaided by the State, other than the minority educational institutions referred to in clause (1) of article 30.”* This has been interpreted to allow affirmative action which could extend to trans athletes as a socially disadvantaged class. Article 15 bars both direct and indirect discrimination by the State. Simultaneously, the article authorises affirmative action for disadvantaged groups to remedy historical disadvantage. The constitutional jurisprudence recognises that formal equal treatment may not achieve substantive equality. Thus, we see the placement of Article 15 (3), Article 15(4) that pave way for targeted measures. The Supreme Court has indicated that the concept of “sex” under Article 15 includes gender and that equality guarantees must be read purposively to curb entrenched social disadvantage. NALSA used these equality guarantees to justify state obligations to transgender persons.

The guardrails of Article 15 would extend to non-discrimination in access to publicly supported sports opportunities. Article 15(1) prevents discrimination in programmes run or funded by the State. We see instances of state scholarships, Sports Authority of India (hereinafter referred to as “SAI”) training camps, reservations for sports quotas in government universities, selection for government-sponsored national teams. Thus, any trans athlete who would be denied a government scholarship, etc., solely because of gender identity would have a *prima facie* claim under Article 15.

The text and purpose of Article 15 permit the State to make special provisions for socially disadvantaged groups. A reasoned policy argument, and one consistent with NALSA's identification of transgender persons as a community facing structural disadvantage, is that trans athletes qualify as a socially and educationally disadvantaged group for the purposes of targeted measures. Applied to sport, this could justify: (a) earmarked scholarships/fellowships for trans athletes; (b) reservation of seats in state sports academies or *Khelo* India cohorts; (c) grant programmes for transgender sports associations to build facilities and mentorship. While Article 15 provides textual and constitutional authority for them, any such measures would have to be proportionate and designed to remedy disadvantage.

Article 15 does not automatically require affirmative measures. It rather confers power on the State to adopt them. For exercise of that power in sport, policy design must identify concrete metrics of disadvantage. Some examples of this can be participation rates, dropout statistics and access to coaching. Accordingly, the governments/federations can tailor programmes to remediate them. Legislative or policy instruments like budget lines in *Khelo* India and NEP Gender Inclusion Fund directed to school-sport, are natural vehicles to operationalize Article 15 (4) in the sporting sphere (10).

#### 2.1.3 Article 16

Article 16 (73) of the Constitution of India secures equal access to public employment, which can apply to sports careers funded by the state. Article 16 ensures non-discrimination and equality of opportunity in state employment. This Article enables reservation as an acceptable instrument to remedy past exclusion. Many sports careers in India are closely intertwined with state employment, because the institutional pathways that sustain athletic participation are largely embedded within government supported structures. Positions in the Sports Authority of India (SAI), roles in national and state sports federations, employment in government sports departments, appointments in public universities, and athletic scholarships frequently function as pipelines to salaried roles offered by various state departments such as coaching, training, and sports administration.

Article 16's non-discrimination mandate therefore extends directly to transgender persons who should not be excluded from these posts or training schemes on the sole basis of gender identity. For example, a rule barring a transgender candidate from a government-sponsored coaching course would trigger a violation of Article 16.

Further, Article 16(4) enables reservation for inadequately represented classes. If policy evidence shows transgender persons are inadequately represented in state sports services or government-run athlete pipelines, legislatures could permit reservation quotas in government sports jobs or reserved slots in state training camps under the Article 16(4) rubric. The same logic supports earmarked recruitment drives for transgender coaches or sports administrators. As with any reservation scheme, the measure must meet constitutional tests on identification of the beneficiary class and proportionality.

#### 2.1.4 Article 21

Turning to the most expansive and powerful shield of constitutional values is the protection under Article 21 (74). Itprotects “life and personal liberty,” a right held to include dignity. Excluding someone from sports solely for their gender identity likely violates dignity and personal liberty. In NALSA, the Court expressly connected gender-identity recognition to Article 21, holding that recognition of one's gender identity is integral to one's right to life and dignity and that denial of such recognition amounts to a violation of Article 21. Later in (11) the Court read Article 21 together with Article 14 and Article 15 to strike down discriminatory criminal rules and to affirm dignity and autonomy of sexual minorities.

In the sports ecosystem, Article 21 protects an athlete's dignity and bodily autonomy against invasive, humiliating procedures. When sports authorities/ federations impose sex verification processes that involve invasive examinations, public disclosure of medical reports, or coercive surgeries or hormone interventions as a precondition for competition, those practices can be challenged under Article 21. The NALSA framework strengthens this claim by providing that gender identity rests within the intimate domain of personal autonomy protected by Article 21. The historical examples of intrusive testing or public exposure of test results raise Article 21 concerns, both for the procedural fairness of testing regimes and for the privacy of medical information.

The right to life with dignity also includes the right to participate in social, cultural and recreational life without discriminatory exclusion. Sport is a form of cultural participation. Exclusion from competitive sport on the ground of gender identity threatens the dignity based right to participate. Judicial recognition of dignity therefore supplies a constitutional basis for contesting blanket exclusions or demeaning procedures that prevent transgender persons from enjoying the social benefits of sport.

### 2.2 International covenants

India is a party to international instruments that enshrine non-discrimination and civil liberties, notably the Universal Declaration of Human Rights (12) and the International Covenant on Civil and Political Rights (ICCPR) (13).

The Yogyakarta Principles (14), though non-binding, are an authoritative statement by human rights experts interpreting how international human rights standards apply to sexual orientation and gender identity. Principle 11 and related principles affirm the right to participate in cultural life without discrimination (14). The Yogyakarta Principles provide a rights-based interpretive framework that emphasises self-identification, the right to participate in public life, and the illegitimacy of forced medical treatment, making its application equally relevant to sporting eligibility rules.

International human rights norms are used by Indian courts to interpret constitutional guarantees. The Supreme Court of India frequently relies on international jurisprudence to elucidate dignity, privacy and non-discrimination. These instruments provide strong normative backing for transgender and intersex inclusion in sports. When crafting sport policy or adjudicating disputes, Indian authorities and courts can therefore rely on these international standards to interpret Article 14, 15, 21 and to insist that any socio-administrative measure is necessarily crafted in a manner to be rights-respecting and affirmatively enabling.

### 2.3 Judicial pronouncements

Several landmark court decisions have shaped the landscape. In *NALSA v. Union of India* (15) the Supreme Court recognized transgender persons as a “third gender,” entitling them to fundamental rights equal to all citizens, including the right to self-identify one's gender. NALSA directed governments to treat trans persons as socially and educationally backward, and to formulate welfare measures.

Conversely, Suresh Kumar Koushal v. Union of India (15) was a setback. It upheld (16) which criminalised gay sex and contained sweeping comments on homosexuality. Koushal was overturned by Navtej Singh Johar judgement (15), but its interim effect was to remind rights advocates that India's legal system can be regressive.

In the sports context, no Indian court directly adjudicated a trans athlete's eligibility until very recently. However, national courts have acted on related issues. For example, the Madras High Court in 2021 in (17), a case of police harassment of trans person) noted how “exposure to what lies beyond heteronormativity is limited” and ordered the government to run sensitivity programs (S. Sushma Case). Although not about sports, this judgment emphasized the stigma faced by trans people. More pertinently, in a 2022 Kerala High Court case (18) a trans girl challenged denial of entry in a judo tournament. The Court held that in the absence of any specific “transgender” category, a trans athlete must be allowed to compete in the category consistent with their gender identity, provided they meet the usual requirements, such as any medical or hormonal criteria the category normally enforces. The Court stated: “*A transgender person has an equal right to participate in competitions. In the absence of any category for participating transgender persons, the petitioner will have to be permitted to participate in her chosen category”*. These judicial statements strengthen the principle that exclusion by default is impermissible (19).

Another important case is (20) which challenges certain provisions of the Transgender Persons (Protection of Rights) Act (21) as unconstitutional for not adequately implementing NALSA. While NALSA judgement of 2014 was groundbreaking, the Parliament's later legislation of Transgender Persons (Protection of Rights) Act (21) has been criticized for betraying NALSA's spirit by not including any affirmative provision for inclusion of transgender persons within public employment or educational sector. It is noted that the 2019 Act transgressed from the provisions of NALSA judgment, and that many of NALSA's recommendations were fundamentally ignored.

### 2.4 Transgender persons (protection of rights) Act 2019 and rules 2020

In 2019, Parliament enacted The Transgender Persons (Protection of Rights) Act (21). The Act's stated aim is to protect trans persons’ rights and prohibit discrimination. Key provisions relating to sports and recreation include:

Section 8 (5) requires the government to “take appropriate measures to promote and protect the right of transgender persons to participate in cultural and recreational activities.”

Section 13 obliges every educational institution to provide “inclusive education and opportunities for sports, recreation, and leisure activities to transgender persons without discrimination on an equal basis with others”. This explicitly makes sports a protected sphere for inclusion.

In addition, the Act broadly prohibits discrimination on the ground of gender identity (Section 3) and mandates rehabilitation and social integration measures (Section 12). It also establishes the National Council for Transgender Persons (NCTP) to advise the government on policy. For trans athletes, the Act's language creates a legal duty on governments and institutions to be inclusive in sports: in principle, sports federations and schools must accommodate trans athletes equally. However, the Act lacks specificity on implementation. Section 13, for instance, while promising inclusivity in educational sports, does not spell out how to classify trans students in competitive sports.

The Transgender Persons (Protection of Rights) Rules, 2020 (75) add some clarity. The rules recognize a person as transgender if their gender identity is different from the sex assigned at birth. They require government bodies to take measures for inclusion of transgender persons. But like the Act, the Rules largely address social welfare, like education, employment, healthcare and do not include sports-specific guidelines.

Thus, as of 2025 there is no Indian sports regulation explicitly detailing how trans athletes are to be treated in competition; such matters have been left to individual federations or courts. With the Indian sports ecosystem undergoing a paradigm shift, it will become regulatory imperative for the government to make sports inclusive.

### 2.5 Education and sports policy initiatives

The Government of India has promoted various schemes for transgender inclusion. However, no such scheme provides targeted measures for the sporting ecosystem. The National Education Policy 2020 is notable for its acknowledgment of gender inclusion. It classifies transgender children as belonging to socio-economically disadvantaged groups and calls for equitable education for them.

The NEP proposes a “Gender Inclusion Fund” to support socially disadvantaged groups including transgender persons, and emphasizes inclusive curricula. Crucially, the NEP advocates integrating sports into all levels of education. By directing the formation of a fund and encouraging sports in schools, the NEP theoretically creates a channel to support transgender children participation in sports. It is plausible that implementing NEP's provisions, for e.g., gender-sensitive Physical Education (PE) teacher training, creating safe sports spaces, etc. can help strengthen inclusion of trans athletes.

On the sports governance side, India's new (22) (pending enforcement Rules) and the (23) (National Sports Policy) 2025 emphasize broad inclusion. For instance, the Khelo India scheme and NSGA talk about grassroots and people's sports movement, but neither explicitly mentions gender diversity. One commentary has pointed out that “these pillars cannot be operationalized in true sense without including gender-diverse athletes” (24). In practice, this means that unless federations and states proactively integrate trans athletes, policy goals of a 'sports culture for all’ will fall short.

### 2.6 State-Level and NGO initiatives

Some states and organizations have pioneered inclusion models. In Tamil Nadu, the government has published educational materials defining LGBTQIA + terms and running awareness programs, but this has not yet translated into sports policy (25). In Kerala, which became the first state with a formal transgender policy, there has been more progress (26). Kerala held India's first transgender sports meet (2017), where 130 trans athletes competed in sprints, long jump, etc. (27). A Writ Petition in Kerala (the judo case mentioned above (18) led the High Court to direct authorities to allow a trans woman athlete to compete as a woman (28). These examples show that proactive measures like organising sports meets, introducing enabling policy directives, etc., can make a difference, though activists note such events should not be one-off competitions but a step toward regular inclusive participation.

Grassroots NGOs have also taken the initiative. In Coimbatore, the Mangaiyanavan Foundation, comprising physical education teachers, runs athletic and martial arts training camps for trans athletes. Its director, Jamuna Rajavelappan has pointed out that Indian sports authorities still lack comprehensive guidelines on transgender participation (29, 30). Competitive sports continue to remain binary. The absence of sustained opportunities forces many novice trans athletes to quit sports at the early stage. In Manipur, a state in North East India, activist Sadam Hanjabam formed *Ya.all* which translates to ‘Revolution’, and organized football matches exclusively for trans players, creating India's first transgender football team. He noted that international federations’ non-recognition of third-gender teams trickles down to local exclusion. His team was barred from official registration and forced to register as a women's team simply because players were “born women” on paper. His appeal is for official recognition and resources (31).

These initiatives highlight the creative responses of civil society. They also underline systemic gaps. Without federation support or legal mandates, inclusion depends on individual activism. Although government schemes exist like PM-DAKSH which is a scheme of skill development for vulnerable groups, and SMILE, a government scheme that provides support for trans persons’ education and livelihood, none have a provision for explicitly funding sports training (32, 33). Such scheme can be beneficial in the sports ecosystem as well. For example, transgender youth could benefit from PM-DAKSH funding to create sports training modules, or SMILE to support mentoring of budding trans athletes and run anti-stigma campaigns. Currently, however, there is no framework or policy initiatives specifically for trans-athletes.

## 3 Discussion on historical and social context

Hindu society traditionally recognized non-binary genders. The *hijra* have been a visible third gender in India for centuries: “[Hindu] society features the gender hijra, the most common nonbinary identity recognized in [India] today. Hijras are found in Hindu religious texts and throughout South Asian history” (3).

The recognition of gender minorities in India has undergone a significant transformation over the past century. While colonial era laws imposed marginalisation and social stigma upon non-binary and transgender communities, the 21st century has witnessed a marked restoration and expansion of rights for these groups (34, 35). India's obligations towards gender equality are buttressed by its endorsement of major international human rights instruments. For instance, India is a signatory to the foundational documents of the Universal Declaration of Human Rights, 1948 (hereinafter referred to as “UDHR”), International Covenant on Civil and Political Rights, 1966 (hereinafter referred to as “ICCPR”). The nation has also embraced the Yogyakarta Principles of 2006, which specifically articulate the rights of individuals regarding sexual orientation and gender identity, setting international standards for the protection and realisation of these rights.

India's domestic legal framework through its Constitution enshrines several equality clauses that are central to the protection of gender minorities including Article 14, 15 and 16. Collectively, these constitutional provisions provide a robust legal foundation for advancing the rights and dignity of gender-diverse individuals in India as elucidated in previous sections. Landmark Supreme Court decisions have further clarified and expanded the rights of gender minorities. Thus, Indian laws and courts technically endorses trans/non-binary inclusion. However, Indian sports governance has lagged.

Most national federations still operate under old ‘women's events only’ rules, leading to conflicts when Indian athletes with intersex traits or transgender identities excel in women's sports. Despite these constitutional and international commitments, the lived realities of non-binary and transgender athletes in India have remained fraught, as demonstrated by recent case studies. While the legal framework provides for equality and self-identification, sports bodies continue to enforce binary rules that exclude or stigmatise athletes whose identities or biology do not conform to traditional categories. This disconnect has resulted in high-profile controversies and personal hardship, with athletes subjected to invasive scrutiny, discriminatory policies, and sometimes outright exclusion from competition.

The following section examines selected Indian case studies to illustrate the lived realities of athletes who do not conform to conventional sex-based sporting classifications. While particular emphasis is placed on the experiences of trans athletes like Anaya Banger and Aryan Pasha, the cases of Dutee Chand, Shanthi Soundararajan and Nandini Agasara are analysed in the next section to demonstrate the distinct yet interconnected challenges faced by gender-diverse and sex-variant athletes in Indian sport. These stories reveal structural barriers, underscoring the urgent need for sports governance in India to align more closely with constitutional values of dignity, bodily autonomy, and substantive inclusion firmly embedded in Indian law.

## 4 Indian athlete case studies

### 4.1 Anaya banger

#### 4.1.1 Background and controversy

Anaya Bangar, the daughter of former Indian cricketer, coach, and commentator Sanjay Bangar. Anaya, prior to transitioning, progressed through Mumbai's men's junior cricket system, representing teams and clubs including Hinckley Cricket Club in Leicestershire (9). She later publicly disclosed her gender transition, including hormone replacement therapy and gender-affirming surgery. Her coming out placed her at the centre of a national debate on transgender inclusion in Indian sport, particularly cricket, bringing issues of eligibility, fairness, and athlete rights into sharp focus.

#### 4.1.2 Issues

Anaya's decision to publicly disclose her transition and release an eight-page athlete testing report asserting her medical eligibility for women's cricket triggered three interconnected points.

*Firstly*, her case exposed regulatory barriers arising from the International Cricket Council's (hereinafter referred to as “ICC”) gender-eligibility framework (36), adopted by the Board of Control for Cricket in India (hereinafter referred to as “BCCI”), and its November 2023 policy (37) excluding athletes who have experienced male puberty from international women's cricket. This categorical rule effectively forecloses elite-level participation for transgender women regardless of hormone therapy or surgical transition and leaves little room for individualised assessment. Anaya's public appeal to the ICC and BCCI was therefore a direct challenge to this rigid regulatory framework and an attempt to reopen space for evidence-based evaluation (38).

*Secondly*, her experience also highlighted the absence of an effective domestic dispute-resolution mechanism for cricket-related eligibility disputes. Unlike athletics, where gender-eligibility cases have been litigated before the Court of Arbitration for Sport (hereinafter referred to as “CAS”), transgender cricketers in India have historically been left to pursue constitutional remedies before national courts or seek relief through international sports law bodies. Although the Sports Governance Act 2025 proposes the establishment of a National Sports Tribunal (hereinafter referred to as “NST”), but under Section 20 (39), its jurisdiction, procedures, and operational framework remain uncertain.

*Thirdly*, Anaya's case revealed the substantial social costs associated with visibility for transgender athletes. She reported experiences of sexual harassment, including unsolicited explicit messages and lewd propositions from within the cricketing ecosystem, highlighting the pervasive culture of misogyny and transphobia that compounds exclusion beyond formal regulation (40).

#### 4.1.3 Why her story matters?

Anaya's case underscores the limits of categorical eligibility rules and highlights the need for individualised, evidence-based assessment frameworks that respect bodily autonomy while addressing fairness concerns. It also draws attention to non-regulatory harms faced by transgender athletes, including social harassment and exclusion, despite the absence of formal disqualification. The significance of her advocacy is amplified by cricket's unique cultural prominence in India, which enabled unprecedented public scrutiny of existing policies and shifted the conversation toward structural reform, including clearer eligibility standards, confidential medical protocols, and institutional safeguards against discrimination.

### 4.2 Aryan pasha

#### 4.2.1 Background and controversy

Aryan Pasha, born in 1991 in Delhi, is an Indian lawyer, activist, and bodybuilder who became the first transgender man in India to place in a men's bodybuilding competition. A former national-level skating champion, Pasha transitioned in early adulthood with family support, commencing hormone therapy at eighteen and later undergoing gender-affirming surgery (41). He shifted from legal practice to bodybuilding, identifying it as a sporting space more closely aligned with his affirmed gender identity and embodied experience of masculinity (42).

Pasha's case emerged as a governance challenge due to the absence of transgender-inclusive eligibility pathways in Indian sport. With limited engagement from international bodybuilding federations and no recognised transgender categories domestically, he elected to compete in the men's division at Musclemania India (43). His participation highlighted the structural exclusion faced by trans athletes, who are often compelled to enter cisgender categories or exit competitive sport altogether.

#### 4.2.2 Issue

Indian bodybuilding, like most sports, lacks formal eligibility frameworks for trans athletes. Pasha's entry into Musclemania India occurred in the absence of institutional guidance or protective policy, relying instead on individual discretion. This exposed a broader regulatory vacuum in Indian sport, where inclusion depends on *ad hoc* decision-making rather than rights-based governance.

As Musclemania India adheres to natural bodybuilding standards prohibiting exogenous hormone use, including testosterone, for Pasha, whose gender affirming care requires regular testosterone administration, this created a direct conflict between medical necessity and sporting regulation. Organisers ultimately allowed him to compete on the condition that he forgo his final testosterone dose prior to competition, an improvised compromise that underscored the lack of clear, evidence-based medical protocols for trans athletes (44).

Beyond formal rules, Pasha's experience reflects persistent non-regulatory harms. He has reported institutional insensitivity and social exclusion both within educational settings and the sporting ecosystem, revealing how stigma and limited understanding of transgender identities continue to shape participation even in the absence of explicit disqualification (42, 44).

#### 4.2.3 Why his story matters?

Pasha secured second place in the Men's Physique (Short) category at Musclemania India, marking a watershed moment for transgender participation in mainstream Indian sport. His achievement demonstrated that transgender men can compete within conventional sporting structures while simultaneously exposing the inadequacy of existing governance frameworks. Subsequently, Pasha has continued to compete in national and international bodybuilding events and has emerged as a prominent advocate for transgender inclusion (45).

His appointment as an expert member of the National Council for Transgender Persons under the Transgender Persons (Protection of Rights) Act (20, 46) situates his sporting experience within a broader rights-based advocacy framework. His advocacy extends to collaborations with civil society organisations engaged in promoting transgender rights, public education, and community empowerment. Alongside prominent transgender activist Laxmi Narayan Tripathi, Pasha co-founded LA Beauté & Style Salon in Ghaziabad, an enterprise employing primarily transgender staff, thereby contributing to the creation of inclusive employment opportunities and challenging systemic labour-market discrimination (47).

His case, like that of Anaya Bangar, illustrates the limits of categorical regulation and the urgent need for transparent eligibility criteria, medical confidentiality, and institutional safeguards that balance inclusion, fairness, and athlete welfare within Indian sport.

### 4.3 Shanthi soundarajan

#### 4.3.1 Context and background

Shanthi Soundarajan, born in 1981 in Tamil Nadu, rose from extreme poverty to become one of India's most promising middle-distance runners. Training barefoot on mud tracks while supporting her family through daily-wage work, she won multiple national medals and achieved international recognition with a silver medal in the 800 m at the 2006 Asian Games in Doha. Her success symbolised resilience and upward mobility through sport (48). This trajectory was abruptly derailed when she was subjected to a gender verification process soon after her success, the outcome of which was never clearly explained to her. She was stripped of her medal, publicly humiliated, and left without institutional support, marking one of the most traumatic episodes in Indian sporting history (48, 49).

#### 4.3.2 Issues and challenges

The challenge was triggered by opaque testing procedures conducted under the authority of the Olympic Council of Asia in coordination with the IAAF medical panel (50) following her Asian Games performance (51, 52). After routine testing revealed anomalies, Shanthi was made to undergo gender verification without informed consent, counselling, or explanation. She was verbally informed that she had failed the test, reportedly due to intersex variations such as androgen insensitivity syndrome, but received no written report or opportunity to contest the finding (51).

Legally, her case exposed severe gaps in sports governance. She was denied access to medical evidence, procedural fairness, and any effective remedy, violating basic principles of natural justice. Indian law offered no protection against discrimination based on sex characteristics, and domestic sports bodies disclaimed responsibility by deferring to international federations (53).

Procedurally and ethically, the testing was invasive, non-consensual, and conducted without confidentiality. Her medical details were leaked to the media, resulting in intense public scrutiny. The absence of counselling, rehabilitation, or legal assistance compounded the harm. Socially, she faced ostracism, loss of livelihood, and a severe mental health crisis, including a suicide attempt (53), followed by years of poverty and neglect. Reflecting on the extent of this social rejection, she later said, “*After the Asian Games I thought, by dressing like a man, there would be greater acceptance*,” a statement that captures both her desperation and the enduring impact of the stigma she had been forced to bear (51).

#### 4.3.3 Why her story matters

Shanthi never recovered her medal, but gradual public recognition led to her appointment as a state athletics coach after nearly a decade. She has since spoken out against unethical sex-testing practices. Her story represents the pre-regulation era of arbitrary gender verification and underscores the profound human cost of opaque, unscientific policies. It remains a powerful reminder that sports governance must prioritise dignity, due process, and scientific integrity, particularly for marginalised athletes.

### 4.4 Dutee chand

#### 4.4.1 Context and background

Born in 1996 in Odisha, Dutee Chand emerged as one of India's most promising sprinters in the 100 m and 200 m events. Coming from a rural and economically marginalised background, she broke national junior records and became the youngest Indian athlete to qualify for the 2013 World Championships (54). By 2014, Chand was widely recognised as “India's fastest woman” (54) and was expected to lead India's sprinting contingent at major international competitions. However, just weeks before an international event, she was abruptly declared ineligible for women's competition (55), marking the beginning of a landmark legal and policy challenge that reshaped global discourse on sex testing in sport (56).

#### 4.4.2 Issues and challenges

Chand was barred from competition under the IAAF's Hyperandrogenism Regulations (57) after tests indicated that her naturally occurring testosterone levels exceeded the prescribed threshold. The policy relied exclusively on hormonal benchmarks, without requiring evidence of actual performance advantage. Chand refused to undergo medically unnecessary corrective procedures to lower her testosterone, citing risks to health, dignity, and bodily autonomy, and instead challenged the regulation before the CAS (58).

The case raised interconnected legal, scientific, and ethical concerns. Legally, it questioned whether excluding women based on innate biological traits amounted to discrimination. Scientifically, the policy lacked robust, peer-reviewed evidence demonstrating a consistent performance advantage linked to endogenous testosterone across events. Ethically, the regulations enabled coercive medicalisation, invasive scrutiny, and privacy violations, disproportionately affecting women from the Global South and reinforcing narrow definitions of womanhood (58).

#### 4.4.3 Why her story matters

In 2015, CAS suspended the Hyperandrogenism Regulations, finding insufficient scientific justification to uphold them (59). Chand was reinstated and returned to competition, later achieving international success, including gold at the 2019 World University Games. Although modified regulations were introduced in 2018, her events remained unaffected.

However, a significant regulatory shift occurred in March 2023, when the World Athletics Council replaced the event-specific framework with a universal eligibility requirement applicable to all track and field events. The revised rules lowered the maximum permitted level of circulating serum testosterone from 5.0 nmol/L to 2.5 nmol/L and required athletes to maintain this level for at least 24 months in order to compete in the female category (60).

In 2025, further amendments introduced mandatory SRY gene testing as part of eligibility verification, thereby embedding compulsory biological screening within the regulatory framework (56). These developments signal a move toward more stringent and standardised classification mechanisms across events, including sprint disciplines such as those in which Chand competed (61).

Chand was later banned by the National Anti-Doping Agency (NADA) for four years in an unrelated matter and has not competed since 2023. Subsequently she announced her retirement following the rejection of her appeal (62). The evolution of World Athletics regulations raises an important counterfactual question. Had she continued her career, the 2023 and 2025 amendments may have required her to confront a significantly altered and more demanding eligibility regime. This underscores how regulatory shifts continue to reshape the landscape first challenged through her landmark case.

Nevertheless, Dutee Chand's case stands as a watershed moment in sports. It shifted the focus from regulating women's bodies to safeguarding bodily autonomy, scientific integrity, and human rights. Her courage established a global precedent against arbitrary exclusion and forced medicalisation, underscoring the urgent need for transparent, evidence-based, and rights-affirming gender eligibility frameworks in sport (6, 59). Chand's struggle extended beyond law into profound personal harm. She endured media outing, privacy violations, and public ridicule that reduced her identity to biological scrutiny (6, 55, 63). Despite this, her resistance transformed global sports, advancing bodily autonomy and establishing a lasting precedent for rights-based, non-discriminatory gender eligibility in sport.

Her story becomes an important landmark in the global debate around hyperandrogenism. It unveils less deliberated upon struggles of global south athletes. While analysing significant case studies such as that of Dutee Chand, recommendations often cover perspectives that might counter lived-experiences of athletes from the global north and ignore unique setups prevailing in the global south (64). It is significant to note that the thread of intersectionality is the foundation of a homogeneous safe sport environment and thus should be upheld while devising an inclusive sport ecosystem (65). It is pertinent to note that research associated with contemporary gender related issues in sports often highlights friction between scholars from the global north and the global south. Therefore, Dutee Chand's case should be placed on a higher pedestal to represent the voices of athletes from global south.

### 4.5 Nandini Agasara

#### 4.5.1 Context and background

Nandini Agasara emerged as one of India's promising young heptathletes after clinching the bronze medal at the 2023 Asian Games (66). Hailing from a modest family in Hyderabad, her achievements serve to be widely celebrated. However, shortly after the event, a social media post by fourth-placed athlete Swapna Barman implied that Nandini was a transgender woman (67), outing her without evidence or official inquiry, and triggering widespread scrutiny.

#### 4.5.2 Issues and challenges

The allegation subjected Nandini to immediate social and psychological stress. She faced humiliation and mental distress as her victory became overshadowed by baseless claims about her gender (37, 68). Institutionally, the Athletics Federation of India (AFI) dismissed the allegation and sought clarification from Barman, who later issued a public apology (37, 68).

Although Nandini did not pursue legal action, the episode raises significant rights-based concerns. Her privacy was violated, her dignity questioned, and her credibility as an athlete undermined. The unfounded claim risked defaming her and illustrates how a single baseless allegation can generate prolonged public scrutiny. Later, speculation resurfaced after her withdrawal from the 2025 World Athletics Championships, though the AFI clarified it was due to an elbow injury, and World Athletics confirmed her eligibility, highlighting the importance of confidential handling of sensitive athlete information (69).

#### 4.5.3 Why her story matters

While the controversy subsided, the incident revealed institutional gaps in communication, athlete protection, and management of sensitive identity-related issues. Nandini's case underscores the dangers of public outing, the need for athlete-centric privacy and anti-harassment safeguards, and the importance of transparent, humane procedures for handling gender, sex, or DSD-related concerns. Despite that that there was a public apology and as remedial measure laws pertaining to defamation exist, her case highlights that, in the absence of robust governance frameworks, even cisgender women can become targets of suspicion, emphasizing the urgent need for rights-based reforms in Indian sport.

## 5 Actionable recommendations

India's engagement with non-binary, transgender, and intersex athletes presents a paradoxical picture of incremental progress coexisting with deep structural inertia. On the one hand, landmark moments, notably the social, legal and sporting battles fought by athletes have compelled institutions to acknowledge gender diversity and confront the arbitrariness of exclusionary practices. These cases have contributed to a growing recognition, both judicial and public, that sporting excellence cannot be mapped onto binary notions of sex. On the other hand, the everyday realities faced by gender-diverse athletes reveal enduring systemic failures that continue to marginalize them within India's sporting ecosystem.

Several critical gaps emerge from the case studies and policy analysis discussed in this paper. The lived experiences examined in this paper reveal that inclusion in Indian sport is shaped less by the absence of rights and more by the failure to operationalize them. Four interrelated deficits define the current landscape. *First*, Indian sports governance suffers from a regulatory vacuum. No national federation has articulated comprehensive eligibility or inclusion guidelines for transgender or intersex athletes, leaving decisions to *ad hoc* discretion. This silence stands in consonance with increasingly restrictive international regimes, such as World Athletics’ 2023 exclusion of post-pubertal transgender women from women's rankings (70), and fails to offer even minimal procedural clarity at the domestic level. *Second*, the legacy of sex verification continues to inform institutional suspicion toward gender-diverse athletes. Although invasive testing practices have formally ended, their epistemic logic persists, as reflected in recurrent demands for athletes to ‘prove’ their gender and in enduring misconceptions about biological sex. *Third*, social stigma and media sensationalism compound exclusion, framing athletic success by gender-diverse athletes as inherently controversial and deterring participation at both elite and grassroots levels. *Fourth*, administrative inertia manifested in inadequate funding, lack of coaching guidance, and the absence of recognized competitive categories further marginalizes transgender athletes within the sporting ecosystem.

These failures are best understood as an implementation gap, not a normative one. India's constitutional guarantees of equality, dignity, and non-discrimination, reinforced by statutory protections and international human rights commitments, already provide a robust foundation for inclusion. What remains lagging is the translation of these principles into sport-specific governance.

Addressing this gap requires a multi-layered response. National sports bodies must adopt clear, evidence based participation guidelines rooted in constitutional jurisprudence, particularly the self-identification principles articulated in the NALSA judgement. Affirmative measures such as targeted scholarships, earmarked funding under *Khelo* India, and pilot of open or gender-inclusive categories at domestic competitions, are constitutionally defensible and normatively necessary to counter structural disadvantage. Equally critical is systematic sensitization of coaches, administrators, and athletes, alongside investments in indigenous sports science research to inform policy with context-specific data rather than conjecture. Policy implications for Indian sports governance ecosystem must therefore include the following:

- National guidelines: The Ministry of Youth Affairs and Sports (MYAS) should mandate all national federations to adopt uniform, rights-based inclusion policies for transgender and intersex athletes.
- Affirmative support: Existing schemes such as *Khelo* India and university games should earmark funding, scholarships, and training slots for gender-diverse athletes.
- Institutional capacity building: Mandatory gender-sensitization modules should be integrated into coaching certification, referee training, and sports administration programs.
- Domestic autonomy: In the absence of inclusive international standards, India should exercise policy autonomy at the national and sub-national levels to pilot inclusive competition models.

The future scholarship should move beyond doctrinal and anecdotal analysis toward empirical, interdisciplinary inquiry. Priority areas include: (i) physiological and performance related studies contextualized to Indian athletes; (ii) longitudinal research on participation and dropout rates among transgender youth in sport; (iii) comparative analyses of inclusive sporting models across jurisdictions in the Global South; and (iv) qualitative research documenting athletes’ lived experiences of governance, stigma, and resilience. Such research is essential to developing evidence-based policies that balance fairness, inclusion, and human dignity.

As India aspires to position itself as a global leader in sport and inclusion, it must move beyond symbolic recognition toward substantive equality in practice. Ultimately, inclusive sport is not merely a question of eligibility thresholds or competitive fairness. It is a measure of democratic belonging. This requires closing the gap between constitutional promise and institutional behaviour through clear eligibility policies, sustained investment in athlete support, and a cultural commitment to dignity. If India seeks to align its sporting ambitions with its constitutional ethos, transgender and intersex athletes must be recognized not as exceptions to be managed, but as equal participants whose dignity and talent enrich the sporting commons. Learning from the journeys of its athletes, India has the opportunity to move from symbolic inclusion to substantive equality in sport.

## Footnotes

^1^*See*, e.g., the canonical tests developed in *State of West Bengal v. Anwar Ali Sarkar*; *See also,* the Supreme Court’s explicit reliance on Article 14 in recognising transgender rights in *National Legal Services Authority v. Union of India*.
